# Incremental value of cardiac magnetic resonance with adenosine stress perfusion in coronary artery disease detection

**DOI:** 10.1186/1532-429X-15-S1-E57

**Published:** 2013-01-30

**Authors:** Eulalia Pereira, Nuno Bettencourt, Nuno D Ferreira, Andreas Schuster, Amedeo Chiribiri, João Primo, Madalena Teixeira, Lino Simões, Eike Nagel, Vasco Gama

**Affiliations:** 1 Cardiology, Centro Hospitalar de Vila Nova de Gaia/Espinho, EPE, Vila Nova de Gaia, Portugal; 2 Kings College London, London, UK

## Background

A multiple-test strategy in often needed in the evaluation of patients (pts) with suspected coronary artery disease (CAD). Cardiac magnetic resonance (CMR) has potential advantages in this context allowing simultaneous evaluation of ventricular function, myocardial perfusion and scar detection with high spatial resolution and no ionizing radiation exposure. We aimed to compare the diagnostic accuracy of adenosine stress CMR myocardial perfusion imaging (CMR-MPI) with exercise treadmill test (ETT) for detection of CAD using invasive fractional flow reserve (FFR) as the reference standard, and to assess the best performing diagnostic algorithm using these tests in patients with suspected CAD.

## Methods

Prospective single-center study with enrollment of symptomatic pts referred to cardiology to suspected CAD assessment. All pts underwent a sequential protocol including: ETT, CMR-MPI and X-ray coronary angiography (XA). The ETT was dichotomously classified and deemed suggestive of CAD if reproduction of clinical symptoms during effort or additional ST-segment depression ≥1 mm. CMR-MPI exams were evaluated by two independent cardiologists blinded to the results of ETT. Functionally significant CAD was defined by the presence of occlusive/sub-occlusive stenosis or FFR measurements <0.8 in vessels >2 mm. Multiples protocols integrating ETT and CMR-MPI were tested using ROC curves.

## Results

80 pts were recruited, 68% male and with mean age of 61 ± 8 years. All pts were symptomatic and had at least one cardiovascular risk factor. CAD was detected in 37 pts (47%). CMR-MPI and ETT showed similar sensitivity for CAD detection (81 vs. 80%), but with higher specificity for CMR-MPI (93 vs. 56%), translating into a greater diagnostic accuracy of CMR-MPI (AUC=0.87 vs. 0.70; PPV=91 vs. 58%). In the best performing protocol (AUC = 0.83), pts with both clinical and electrically positive ETT would be directly referred to XA while all the remaining pts (negative, borderline positive or inconclusive ETT) would be managed according to CMR-MPI results. The other protocols, including referral to CMR-MPI only the pts with borderline positive or inconclusive ETT or with negative or inconclusive ETT had worst performances (AUC=0.75 and 0.73, respectively).

## Conclusions

CMR-MPI has high sensitivity and specificity for detection of obstructive CAD. In our pts with high probability of CAD, the inclusion of CMR-MPI in an integrated protocol for the detection of CAD improved the overall diagnostic accuracy, particularly in those pts with negative, borderline positive or inconclusive ETT.

## Funding

No conflict of interests/financial disclosures.

